# Subclinical infection without encephalitis in mice following intranasal exposure to Nipah virus-Malaysia and Nipah virus-Bangladesh

**DOI:** 10.1186/1743-422X-11-102

**Published:** 2014-06-02

**Authors:** Johanna Dups, Deborah Middleton, Fenella Long, Rachel Arkinstall, Glenn A Marsh, Lin-Fa Wang

**Affiliations:** 1 CSIRO Animal, Food and Health Science, Australian Animal Health Laboratory, Geelong, VIC 3219, Australia; 2 Duke-NUS Graduate Medical School, 21 Lower Kent Ridge Road, Singapore 119077, Singapore

**Keywords:** Henipavirus, Hendra virus, Nipah virus, Encephalitis, Mouse, Mice, Model

## Abstract

**Background:**

Nipah virus and Hendra virus are closely related and following natural or experimental exposure induce similar clinical disease. In humans, encephalitis is the most serious outcome of infection and, hitherto, research into the pathogenesis of henipavirus encephalitis has been limited by the lack of a suitable model. Recently we reported a wild-type mouse model of Hendra virus (HeV) encephalitis that should facilitate detailed investigations of its neuropathogenesis, including mechanisms of disease recrudescence. In this study we investigated the possibility of developing a similar model of Nipah virus encephalitis.

**Findings:**

Aged and young adult wild type mice did not develop clinical disease including encephalitis following intranasal exposure to either the Malaysia (NiV-MY) or Bangladesh (NiV-BD) strains of Nipah virus. However viral RNA was detected in lung tissue of mice at euthanasia (21 days following exposure) accompanied by a non-neutralizing antibody response. In a subsequent time course trial this viral RNA was shown to be reflective of an earlier self-limiting and subclinical lower respiratory tract infection through successful virus re-isolation and antigen detection in lung. There was no evidence for viremia or infection of other organs, including brain.

**Conclusions:**

Mice develop a subclinical self-limiting lower respiratory tract infection but not encephalitis following intranasal exposure to NiV-BD or NiV-MY. These results contrast with those reported for HeV under similar exposure conditions in mice, demonstrating a significant biological difference in host clinical response to exposure with these viruses. This finding provides a new platform from which to explore the viral and/or host factors that determine the neuroinvasive ability of henipaviruses.

## Introduction

Nipah virus (NiV; genus *Henipavirus*, family *Paramyxoviridae*) is closely related to Hendra virus (HeV; genus *Henipavirus*, family *Paramyxoviridae*) [1] and both cause severe and often fatal encephalitic disease in humans [2,3]. We recently reported a wild-type mouse model of HeV encephalitis [4]; a particularly useful model for this aspect of the disease as, in contrast to many other animal models, mice neither develop systemic infection nor succumb to acute disease prior to establishment of encephalitis. In addition there is excellent access to reagents for more detailed investigation of the brain disease in this species. We were interested to determine whether a similar model of NiV encephalitis in the mouse could be developed given that both viruses use the same host receptor [5,6] and are observed to cause similar clinical disease in several species (reviewed in [7] and [8]).

Nipah virus infection of mice has been investigated previously [9,10]. These studies showed that young adult wild-type mice exposed to Nipah virus using the intranasal or intraperitoneal route did not develop clinical disease. However, effects of mouse strain and age were not described and neither was subclinical infection, which is now known to occur regularly in young adult mice after intranasal HeV exposure [4]. Susceptibility of mice to the Bangladesh strain of NiV has not been reported.

Here we investigate NiV strains from Malaysia (NiV-MY) and Bangladesh (NiV-BD) in infection of young adult and aged mice of two strains (BALB/c and C57BL/6). Both these mouse strains are known to be susceptible to the closely related Hendra virus, with aged mice reliably developing clinical disease [4].

## Experimental design, methods and findings

To study NiV infection in mice, we exposed young adult (8 weeks) and aged (12 months) BALB/c and C57BL/6 mice to 50,000 TCID_50_ of low passage human isolates of NiV-MY (Nipah virus/Malaysia/human/99) or NiV-BD (Nipah Bangladesh/human/2004/Rajbari,R1) by the intranasal route (BALB/c n = 5 all groups, C57BL/6 n = 4 all groups except young adult NiV-BD n = 5). Mice were monitored daily and euthanased at onset of clinical signs or at 21 days post challenge. All animal work was approved by the CSIRO Australian Animal Health Laboratory Animal Ethics Committee.

All mice remained free of clinical disease for the period of observation and were euthanased at day 21. However, consistent with an adaptive immune response to viral replication, many mice were positive for specific binding antibody to the soluble form of NiV G glycoprotein (NiV sG) as detected by Luminex microsphere assay [11] (Table 1). Neutralising antibodies were not detected by serum neutralisation assay.

**Table 1:** Assessment of specific (binding) antibody to NiV soluble-G and neutralising antibody at day 21 post-exposure

**Virus strain**	**Mouse strain**	**Age**	**Mouse #**	**Binding antibody to NiV sG^**	**Serum neutralisation**
Nipah Malaysia	BALB/c	YA*	1	+	-
			2	+	-
			3	+	-
			4	+	-
			5	+	-
		Aged	6	+	-
			7	-	-
			8	+	-
			9	+	-
			10	+	-
	C57BL/6	YA	11	+	-
			12	+	-
			13	+	-
			14	+	-
		Aged	15	+	-
			16	+	-
			17	-	-
			18	+	-
		
Nipah Bangladesh	BALB/c	YA	19	+	-
			20	+	-
			21	+	-
			22	+	-
			23	-	-
		Aged	24	-	-
			25	+	-
			26	+	-
			27	+	-
			28	+	-
	C57BL/6	YA	29	-	-
			30	-	-
			31	+	-
			32	-	-
			33	+	-
		Aged	34	-	-
			35	-	-
			36	-	-
			37	-	-

^sG = soluble-G, *YA = Young adult, for binding antibody to NiV sG (+) was defined as a four fold or greater increase in median fluorescence intensity from pre-challenge levels, for serum neutralisation (−) indicates that no neutralising antibody was detected.

**Figure 1:**
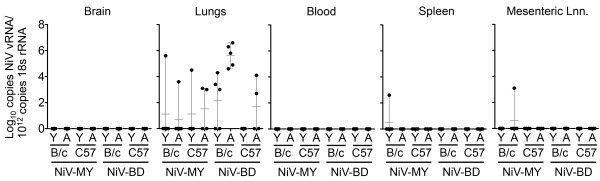
**Nipah viral genome loads in tissue samples 21 days post exposure.** Lungs, blood, spleen and mesenteric lymph nodes (Mesenteric Lnn.) were collected 21 days post intranasal exposure of young adult (Y, 8 weeks of age) and aged (A, 12 months of age) BALB/c (B/c) and C57BL/6 (C57) mice to either Nipah Virus Malaysia (NiV-MY) or Nipah Virus Bangladesh (NiV-BD). Nipah viral RNA was extracted from tissue samples and analysed in triplicate using qPCR assay detecting Nipah viral nucleocapsid protein RNA and 18S rRNA Grey error bars visualise range and mean of data.

To explore the possibility of subclinical NiV infection, we analysed brain, lung, heart, spleen, liver, kidney, mesenteric lymph nodes, ovaries and blood collected at euthanasia (day 21) for presence of viral RNA (vRNA) by real time PCR [12], lesions by histopathology and viral antigen by immunohistochemistry. We attempted virus isolation on all tissues positive for vRNA. All analyses were performed as previously described [4].We did not detect NiV vRNA (Figure 1), viral antigen, or lesions of encephalitis in any mouse brain. However, lung tissue of several mice tested positive for NiV-MY and NiV-BD vRNA (Figure 1), without pulmonary lesions or demonstrable viral antigen. BALB/c aged mice exposed to NiV-BD had higher levels of viral genome in lungs (quantified by comparison to a standard curve to calculate copy number) than any other groups (Figure 1). Remaining tissues were negative for all tests described above except for the detection of vRNA in two cases only (Figure 1), suggesting that systemic infection had not occurred. Of note, these two mice were not positive for vRNA in lung tissue.

To further assess whether the NiV vRNA detected in lungs at 21 days reflected an earlier self-limiting respiratory tract infection, we performed a time course study in aged BALB/c mice exposed intranasally to 50,000 TCID_50_ NiV-MY or NiV-BD. Two mice for each virus strain were euthanased at 48 hour intervals up to day 12 post exposure and at day 15 post exposure.

As expected, animals did not develop clinical disease and were euthanased at the pre-determined time points. Samples were collected and analysed as described above.

**Figure 2:**
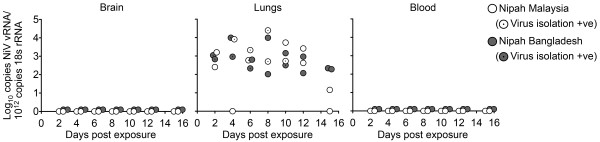
**Nipah viral genome loads and virus isolation from brain, lungs and blood days 0–15 post-exposure.** Twelve month old BALB/c mice were intranasally exposed to either Nipah Virus Malaysia (white circles) or Nipah Virus Bangladesh (grey circles). Two mice per viral strain were euthanased and sampled at 48 hour intervals up to day 12 post exposure and at day 15 post exposure, as indicated (Days post exposure). Nipah viral RNA was extracted from tissue samples and analysed in triplicate using qPCR assay detecting Nipah viral nucleocapsid protein RNA and 18S rRNA. Virus isolation was attempted on all tissues positive for viral RNA. Successful isolations are indicated by a black point (.) within the circles.

**Figure 3:**
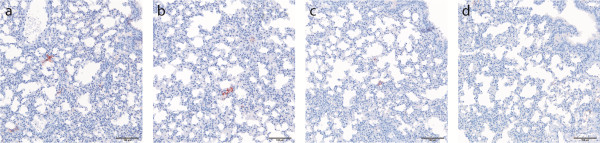
**Nipah viral antigen staining in lungs of intranasally challenged aged mice. a)** NiV-BD and **b)** NiV-MY antigen in mouse lung at 8 dpi during virus replication phase; **c)** detection of small amount of NiV-B antigen at 15 dpi and **d)** clearance of NiV-M antigen by dpi 15 in mouse lung (anti-Nipah N protein).

We detected viral RNA in lung tissue at levels ranging from 1.2 – 4.4 log_10_ copies/10^12^ copies 18 s rRNA at all sampled time points for both virus strains (Figure 2). NiV-MY and -BD were isolated inconsistently from RNA-positive lung tissues using the method described previously (4). To increase test sensitivity 100 ul of supernatant from tissue homogenate was used to inoculate a sub-confluent well of Veros (6 well plate) and re-isolation was successful up to day 10 post challenge (Figure 2). Viral titres were consistently low (<40 TCID_50_/ml). Viral antigen was detected by immunohistochemistry in the alveolar walls of mice infected with NiV-BD and NiV-MY (Figure 3), with mice given NiV-BD significantly more likely to show a positive reaction (p = 0.04, Fisher’s exact test): it was not possible to distinguish on morphologic grounds whether alveolar lining cells, alveolar interstitium, or endothelial cells were involved. Pneumonia was not identified in any mouse. Viremia was not detected at any time point and all remaining tissues including brain were negative for vRNA, viral antigen and viral genome, except for ovary, thymus and heart of one NiV-MY challenged mouse and cervical lymph nodes of two NiV-BD challenged mice which were positive for viral genome but not for antigen nor for lesions.

To test for infection in the upper respiratory tract we examined the nasal cavity of each mouse for evidence of local rhinitis and presence of NiV antigen by histopathology and immunohistochemistry. Viral antigen and lesions consistent with rhinitis were not detected except in one NiV-BD study animal euthanased 10 days post-exposure.

Taken together, these results demonstrate that mice exposed to 50,000TCID_50_ NiV-BD or NiV-MY develop a subclinical, self-limiting lower respiratory tract infection without encephalitis or systemic infection.

## Conclusion and discussion

The observation of sub-clinical self-limiting lower respiratory tract infection without encephalitis in wild-type mice exposed to NiV-MY and NiV-BD contrasted with certain reported findings using HeV in mice under similar exposure conditions [4]. In that report, aged BALB/c and C57BL/6 mice reliably developed neurological disease following intranasal exposure to HeV, with sub-clinical encephalitis also documented in young adult mice. In addition, NiV-MY and -BD were re-isolated from RNA-positive lung tissues less consistently compared to HeV using the method described previously and at lower titres, with levels of HeV reported up to 200 TCID_50_/ml. Considered together, the data suggest that – unlike what has been observed in other permissive animal hosts – NiV infection of mice is less productive than HeV and there is a categorical difference in the pathogenicity of HeV and NiV for mice. In particular, NiV appears unable to establish infection of the central nervous system via the olfactory receptor neuron pathway as observed for HeV. Future *in vitro* studies characterising infection dynamics of NiV and HeV in specific cell types of the olfactory epithelium will help to identify the mechanisms involved in inhibition of NiV neuroinvasion.Increased levels of viral genome were detected in the lungs of aged BALB/c mice exposed to NiV-BD at 21 days compared to other groups (Figure 1). This may be explained by increased virus production during the replication phase of infection or a decreased ability to clear residual viral material. We found comparable levels of viral genome during the period in which replication was observed (up to day 10 post exposure), in aged BALB/c mice following exposure to either NiV-BD or NiV-MY (Figure 2) ruling out the first explanation. The possibility of less efficient clearance of NiV-BD by aged BALB/c mice requires further investigation.

Nipah virus infection detected in lung tissue appeared controlled at this location and did not spread systemically, as viremia was not detected and there was no evidence for productive infection of other systemic organs. Occasional tissues outside of lung were positive for viral genome but not for virus isolation, presence of antigen or presence of lesions and accordingly interpreted as not infected. Control and resolution of the lower respiratory tract infection was not the result of the development of neutralizing antibodies as these were not detected in any mouse at 21 days post-exposure. Similar to HeV, non-neutralising binding antibodies against NiV sG were detected by Luminex microsphere assay, however, their role in control or clearance of infection is unclear and requires further investigation.

Comparing to that observed with HeV in mice [4], the combined infection models elicit a significant biological difference between HeV and NiV at the host level. Under matching conditions and unlike HeV, exposure to NiV lead to infection but not to encephalitis in mice and, as the same mouse strains of the same ages were used in the two studies, it is likely that viral factors (of which dose is one possibility) rather than host factors are responsible for this. Neuroinvasion progressing to encephalitis is the most serious outcome of human infections with henipaviruses: the susceptibility of mice to HeV encephalitis but not NiV encephalitis at equivalent dose presents a key that can be used to define the determinants of henipavirus neuropathogenesis in mice. This information will likely have implications for the development of therapies that can be applied to manage the important neurological complications of human infections.

## Competing interests

The authors declare that they have no competing interests.

## Authors’ contributions

JD conceived the study, carried out the animal infection trials and sampling, processed and analysed samples and drafted the manuscript. DM conceived the study, participated in study design, analysed samples and helped to draft the manuscript. FL carried out histopathological and immunohistochemical analyses. RA carried out the animal infection trials. GM conceived of the study, participated in design of the study and helped to draft the manuscript. LW conceived of the study, participated in design of the study and helped to draft the manuscript. All authors read and approved the final manuscript.
